# Self-Help in Institutional Economy

**Published:** 1912-12-21

**Authors:** 


### SELF-HELP IN INSTITUTIONAL ECONOMY.

### II.—The Meat Supply: A Suggestion.

The amount of meat required for daily consumption in a large hospital is directly proportional to the number of individuals who have to be fed by the institution. It is true enough that the meat ration, for patients as well as for personnel, is larger than it ought to be in most institutions, but it is impossible to change the custom. Even with the strictest economy the total amount of meat necessary for the feeding of a large institution is comparatively large.

Most institutions, like the large shipping firms and hotels, find it convenient to contract, by tender, for their meat supply. In such cases the contractor undertakes to deliver meat, both red and white, in specified quantities, more or less bone free, and prepared more or less ready for the kitchen, such meat to be up to a certain standard. It is, however, a well-known fact that our hospitals possess no means of enforcing these conditions. The meat that is delivered by contractors is butchers' meat, the meat of animals slaughtered not in well-managed abattoirs under expert supervision, but in private slaughter-houses often, as has been pointed out more than once, under conditions of the worst possible kind. No bacteriological inspection of such meat is made as a matter of routine. The hospital authorities have even no means of knowing whether the meat delivered to them as "home grown" is really so or whether it is the meat of foreign animals which, brought alive to an English port, are there killed. The difference from a dietetic point of view between such meat and real home-grown meat is very marked, but before the joint appears on the table there is no means of telling the one from the other. Yet another point of interest is that institutions which contract for their meat lose a great deal of money in two ways: they have to pay the market prices for killed meat, and they lose the profits to be derived from the sale of skins, blood, and other by-products, profits which are often considerable.

### The German "Meat Kitchen."

These considerations have induced the German hospitals, which consume relatively more meat per head than do English institutions, to deal with the meat supply in a more direct manner. The first attempt was obviously to buy whole, skinned carcases, and to utilise such portions of these as were unsuitable for joints, etc., in the preparation of sausages, minced meat, extracts, etc. This has led to the "meat kitchen," which is a feature in every large modern German hospital, and of which the best examples are seen at the Munich hospitals and at the Virchow Hospital at Berlin. The meat kitchen is in charge of an expert butcher, and is provided with adequate cold-storage accommodation and a cool chamber which permits the joints to be "hung" for some time. Owing to the excellent measures taken by the State for the safeguarding of the public, in the shape of proper bacteriological and veterinary examination at every slaughter-house, the butchers' meat may be relied on, and institutional examination is therefore not required. Notwithstanding this, if the meat is delivered by contractors, a sample of it is subjected to examination as a matter of routine. The sausages and minced meat made in the institutional meat kitchen can therefore be absolutely relied upon.

Some hospitals have progressed further and have eliminated the middleman—the butcher. Owing, again, to the provision of public abattoirs, which permit anyone to be his own butcher, they have been enabled to do this with profit on a large scale. The method employed by these institutions may be illustrated by the example of the large municipal hospital at Düsseldorf. This hospital buys at auction so many cows, oxen, sheep, pigs, and calves as are considered necessary. An experienced butcher, cattle buyer, and veterinary surgeon superintend the sales, which are conducted in the local cattle market at stated intervals, and the purchases are made in competition with local butchers. Usually enough live meat is purchased to last the institution for a week. The animals are slaughtered at the public abattoir (formerly by arrangement with private slaughterers), and all the "offal" is sold. Thus the hides, hoofs, and hair are disposed of by auction; the blood is contracted for, and the intestines are carefully overhauled and cleaned for use in the institution itself. The hospital is a member of the local butchers' guild, and enjoys the full privileges secured by such membership; the greatest cordiality prevails between the institution and the local butchers, and although at first the inevitable cry of "unfair competition" was raised, it soon died down.

That economy thus has been secured there can be no question. The initial expenses were of course heavy. Special cool rooms and cold-storage chambers had to be added to the economic department of the kitchen; a special staff had to be engaged not only for the practical work in connection with the dressing of the carcases, but also to keep the accounts of the department (which, as it is a commercial department, even though it does not sell its products, have to be kept separate from the ordinary institutional accounts); and, finally, certain commissions had to be paid. In addition, the institution has had to make provision to meet contingencies. Of these, the most common is loss due to disease discovered in the animals after they have been slaughtered. The carcases of animals with tubercular mesenteric glands are of course destroyed—a direct loss, against which the hospital has effected a special insurance. It is also insured against loss due to failure of the cool storage and to loss incurred by accident. Nevertheless, the new department decreased the butcher's bill in its first year by nearly 30 per cent. Since the decrease has been progressive and for the last two years the department has actually shown a small excess of income over expenditure. Several German hospitals possess private "piggeries" also.

